# Impact of Alcohol Abuse on the Adaptive Immune System

**DOI:** 10.35946/arcr.v37.2.04

**Published:** 2015

**Authors:** Sumana Pasala, Tasha Barr, Ilhem Messaoudi

**Affiliations:** Sumana Pasala, Ph.D., is a postdoctoral fellow; Tasha Barr is a graduate student researcher; and Ilhem Messaoudi, Ph.D., is an associate professor in the Division of Biomedical Sciences, School of Medicine, University of California, Riverside, Riverside, California.

**Keywords:** Alcohol use, abuse and dependence, alcohol use disorder, heavy drinking, beneficial moderate alcohol consumption, ethanol consumption, prenatal alcohol exposure, alcoholic liver disease, immune system, adaptive immune system, immune response, growth and development, infection, T cells, B cells, lymphocyte, immunoglobulin, vaccinations, cancer, pneumonia, HIV, hepatitis C virus, tuberculosis, human studies, animal models

## Abstract

Alcohol exposure, and particularly chronic heavy drinking, affects all components of the adaptive immune system. Studies both in humans and in animal models determined that chronic alcohol abuse reduces the number of peripheral T cells, disrupts the balance between different T-cell types, influences T-cell activation, impairs T-cell functioning, and promotes T-cell apoptosis. Chronic alcohol exposure also seems to cause loss of peripheral B cells, while simultaneously inducing increased production of immunoglobulins. In particular, the levels of antibodies against liver-specific autoantigens are increased in patients with alcoholic liver disease and may promote alcohol-related liver damage. Finally, chronic alcohol exposure in utero interferes with normal T-cell and B-cell development, which may increase the risk of infections during both childhood and adulthood. Alcohol’s impact on T cells and B cells increases the risk of infections (e.g., pneumonia, HIV infection, hepatitis C virus infection, and tuberculosis), impairs responses to vaccinations against such infections, exacerbates cancer risk, and interferes with delayed-type hypersensitivity. In contrast to these deleterious effects of heavy alcohol exposure, moderate alcohol consumption may have beneficial effects on the adaptive immune system, including improved responses to vaccination and infection. The molecular mechanisms underlying ethanol’s impact on the adaptive immune system remain poorly understood.

In the United States, alcohol use disorder (AUD) is the third-leading cause of preventable death. It is associated with increased susceptibility to bacterial pneumonia; viral infections, such as HIV and hepatitis C virus (HCV); and increased postoperative morbidity and mortality. This increased susceptibility is mediated in part by functional alterations in various cells of the immune system. The immune system is broadly divided into two branches: innate and adaptive immunity. The innate immune system represents the first line of host defense and is necessary for inducing the adaptive immune response. The adaptive immune system can be subdivided further into cellular and humoral immunity. The main components of cellular immunity are CD4 and CD8 T cells. CD4 T cells play a critical role in the activation and differentiation of macrophages, CD8 T cells, and B cells. CD8 T cells, on the other hand, are essential for eliminating cells infected with intracellular pathogens, as well as cancer cells. Humoral immunity is mediated by B cells, which produce antibodies to eliminate extracellular microorganisms and prevent spread of infections. This review will summarize the impact of chronic heavy drinking or AUD as well as of moderate alcohol consumption on adaptive immunity and discuss future areas of research in this rapidly evolving field.

## Impact of AUD on T Cells

### Effects on T-Cell Numbers, Phenotype, and Activation

T cells constitute a diverse population of lymphocytes that develop in the bone marrow and mature in the thymus. Each T cell expresses a unique T-cell receptor (TCR) that confers specificity for one particular foreign molecule (i.e., antigen). Early studies already had indicated that chronic alcohol abuse (i.e., for 12 to 15 years) resulted in reduced numbers of peripheral T cells (Liu 1973; McFarland and Libre 1963). More recent studies confirmed this observation and showed that the lack of lymphocytes (i.e., lymphopenia) was as severe in people who engaged in a short period of binge drinking as it was in individuals who drank heavily for 6 months (Tonnesen et al. 1990). Interestingly, abstinence for 30 days was sufficient to restore lymphocyte numbers back to control levels (Tonnesen et al. 1990). Similar findings were obtained in animal models, where the number of T cells in the spleen decreased in mice fed a liquid diet (i.e., Lieber-DeCarli diet) containing 7 percent ethanol for as little as 7 days (Saad and Jerrells 1991) or 6 percent ethanol for 28 days (Percival and Sims 2000). Likewise, adult male Sprague-Dawley rats consuming liquid diets containing up to 12 g ethanol/kg/day for 35 days exhibited significantly reduced absolute numbers of T cells (Helm et al. 1996).

In addition to reducing T-cell numbers, chronic alcohol exposure disrupts the balance between different T-cell types (i.e., T-cell homeostasis), leading to a shift toward a memory phenotype. Specifically, people who had consumed 30.9 ± 18.7 alcoholic drinks/day for approximately 25.6 ± 11.5 years exhibited a decreased frequency of naïve (i.e., CD45RA^+^) CD4 and CD8 T cells, as well as an increased frequency of memory T cells (i.e., CD45RO^+^) (Cook et al. 1994). Another study conducted in humans with self-reported average alcohol consumption of approximately 400 g/day also found an increase in the percentage of both CD45RO^+^ memory CD4 cells and CD8 cells (Cook et al. 1995). These observations were confirmed in animal models. Thus, studies in C57BL/6 mice demonstrated that chronic ethanol consumption (20 percent ethanol in water for up to 6 months) decreased the frequency of naïve T cells and increased the percentage of memory T cells (Song et al. 2002; Zhang and Meadows 2005). This loss of naïve T cells could result from decreased T-cell production in the thymus; increased cell death (i.e., apoptosis) of naïve T cells; or increased homeostatic proliferation. Additional analyses detected evidence that T-cell proliferation in the spleen was increased in alcohol-consuming mice (Zhang and Meadows 2005). Together, these observations suggest that chronic alcohol consumption results in lymphopenia, which can increase homeostatic proliferation and accelerate conversion of naïve T cells into memory T cells (Cho et al. 2000).

Alcohol consumption also influences T-cell activation both in humans and in mouse models (Cook et al. 1991, 1995). For example, alcoholics who had consumed approximately 23 ± 9 drinks/day for 27.0 ± 11.5 years exhibited significantly elevated numbers of activated CD8 T cells immediately after admission for detoxification, which persisted after 4 to 10 days of abstinence (Cook et al. 1991).^1^ The percentage of activated CD8 T cells expressing both human leukocyte antigen (HLA)-DR and CD57 also was increased in alcoholics with self-reported average alcohol consumption of approximately 400 g/day (Cook et al. 1995). An increase in these cells could contribute to the chronic inflammation observed in alcoholic patients, because human CD57-expressing T cells can rapidly produce the pro-inflammatory cytokine interferon gamma (IFN-γ) after stimulation through their TCR, without requiring a second signal, as is the case with other cells (Song et al. 2001). Similar findings were described in mouse models. Thus, C57BL/6 or BALB/c mice that consumed 20 percent ethanol in water for up to 6 months showed a greater frequency of activated T cells, increased rapid IFN-γ response, and heightened sensitivity to low levels of TCR stimulation, with no requirement for a second signal (Song et al. 2002; Zhang and Meadows 2005).

The effects of chronic alcohol exposure are not limited to phenotypic changes in T cells but also include T-cell functions. One study in mice investigated the impact of ethanol or wine consumption on T-cell migration by dividing the animals in three groups (i.e., receiving water, 6 percent ethanol in water, or alcohol in the form of wine adjusted to 6 percent ethanol with water) and injecting them with bacterial molecules called lipopolysaccharides (LPS) or endotoxin, which can activate the immune response. Among other reactions, LPS injection normally triggers lymphocyte migration out of the circulation and into tissues and the lymphatic system (Percival and Sims 2000). In water- or wine-consuming mice, LPS injection, as expected, led to a 50 percent reduction in the number of lymphocytes in the peripheral blood, indicating their mobilization into tissues. In contrast, the ethanol-consuming mice exhibited no change in the frequency of certain circulating lymphocytes (i.e., CD3 cells) after LPS injection, suggesting that chronic alcohol consumption may potentially impair the ability of lymphocytes to migrate out of circulation (Percival and Sims 2000). One potential explanation for the lack of detrimental effects of wine in this experiment could be the presence of phytochemicals in wine that may be able to overcome ethanol’s harmful impact on immunity.

### Effects on T-Cell Apoptosis

Activated T cells normally undergo apoptosis if they receive a second activation stimulus within a short interval. This process is known as activation-induced cell death (AICD) and is important to maintain T-cell homeostasis and self-tolerance (Alderson et al. 1995). Experiments done in an immortalized line of human T lymphocyte cells used in cancer research (i.e., Jurkat cells) found that exposure to different concentrations of ethanol (i.e., 25, 50, 100, 150, 200 mM) for 24 hours resulted in decreased cell viability in a dose-dependent manner. Furthermore, ethanol exposure decreased expression of the anti-apoptotic molecule Bcl-2 and promoted expression of the pro-apoptotic molecule BAX in the cells. These findings suggest that ethanol pretreatment can sensitize T cells to AICD (Kapasi et al. 2003). Similarly, in vitro exposure of peripheral T cells to a physiologically relevant concentration of 25mM ethanol significantly enhanced the activation of a protein that mediates apoptosis (i.e., caspase-3) as well as promoted DNA fragmentation (which is a hallmark of apoptosis) when the cells were stimulated (Kelkar et al. 2002). In vivo studies in humans confirmed these observations, demonstrating that binge drinking (i.e., consuming 5 to 7 drinks within 90 to 120 minutes) promoted T-cell apoptosis and decreased Bcl-2 expression (Kapasi et al. 2003).

Another mechanism contributing to ethanol-induced apoptosis in human T cells could involve down-regulation of the vitamin D receptor (VDR). VDR normally reduces expression of a signaling molecule called renin angiotensin (RAS) (Li et al. 2004). Lowered RAS levels in turn induce dysregulation of the mitochondria (Kimura et al. 2005) and enhance production of reactive oxygen species (ROS) that can damage various molecules in the cells (Iuchi et al. 2003). Both mitochondrial dysregulation and ROS production promote apoptosis. Naïve human T cells produce low levels of VDR, but expression is increased to moderate levels in activated T cells (Irvin et al. 2000). Human T cells incubated in vitro with variable concentrations of ethanol (0, 10, 25, and 50mM for 24 hours) showed a reduced expression of the VDR, accompanied by increased expression of RAS and ROS as well as increased T-cell death (Rehman et al. 2013). Additional analyses demonstrated that ethanol exposure promoted apoptosis by inducing breaks in the DNA of the T cells. This damage to the DNA most likely was mediated by ROS generation in response to RAS activation. Treatment with a compound that activates the VDR (i.e., a VDR agonist) restored the T cell’s VDR expression, down-regulated RAS expression as well as ROS generation, and thus preserved T-cell survival (Rehman et al. 2013).

**Figure 1:**
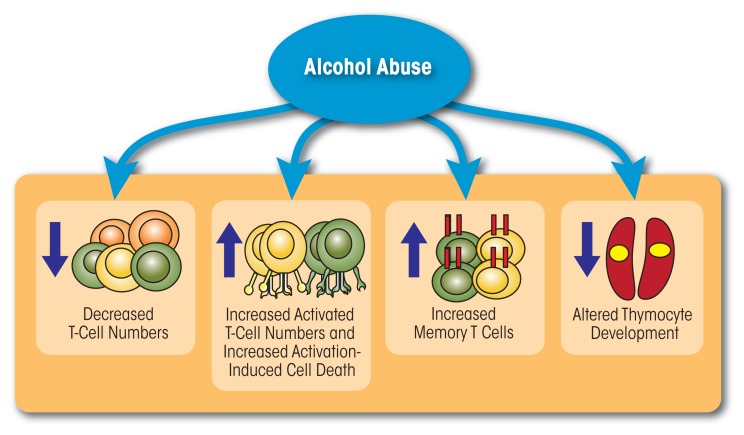
Alcohol abuse affects both the number and function of T cells. Chronic alcohol consumption decreases the number of circulating T cells, increases the number of activated T cells, accelerates differentiation of T cells to a memory phenotype, and interferes with thymocyte development.

In summary, these studies suggest that chronic alcohol abuse in humans and animal models results in lymphopenia, increased T-cell differentiation and activation, and reduced migration (see figure 1). Chronic activation of the T-cell pool may alter the T cells’ ability to expand and respond to pathogenic challenges (potentially by inducing a state of unresponsiveness, or anergy, of the T cells), place the T cells under increased regulatory control, or lead to their elimination through increased sensitivity to AICD. These changes in turn compromise the organism’s ability to respond to pathogens and contribute to increased susceptibility to infections.

## Impact of AUD on B cells

### Effects on B-Cell Numbers and Phenotype

B cells are lymphocytes that originate in the bone marrow and mature in the spleen. They produce proteins called immunoglobulins (Igs) located either on the cell surface (where they are referred to as B-cell receptors [BCRs]) or secreted in the form of antibodies. Like T cells, each B cell expresses a unique BCR that only binds to a specific antigen. B cells and the antibodies they secrete mediate both T-cell–dependent and T-cell–independent immune responses, depending on the specific class of antibody released. Similar to what has been observed for T cells, alcoholics (90 to 249 drinks/month) exhibit lower B-cell numbers than do moderate (30 to 89 drinks/month) or light drinkers (<9 drinks/month) (Mili et al. 1992; Sacanella et al. 1998). The loss of circulating B cells is particularly severe in patients with alcoholic liver disease (ALD) consuming 164.9 to 400 grams of alcohol/day on average (Cook et al. 1996; Matos et al. 2013).

The loss of peripheral B cells primarily seems to affect certain subpopulations of cells. B cells can be divided into two main subtypes that produce different types of Igs and other proteins and respond to particular types of antigens:

- B-1 B cells, which primarily seem to respond to polysaccharide antigens, such as bacterial LPS. They can be divided further into the B-1a subset, which produces broadly reactive IgM antibodies, and the B-1b subset, which is important for T-cell–independent responses.
- B-2 B cells, which are considered “conventional” B cells. They produce high-affinity antibodies and, unlike B-1 B cells, B-2 B cells can develop into long-lived memory B cells that are critical in protection from subsequent infection with the same pathogens (i.e., form an immunological memory).

The alcohol-related decrease in peripheral B cells primarily seems to be mediated by a decrease in the frequency of the B-2 B cells. The number of B-1a cells also seems to decline, but this decrease is accompanied by a relative increase in the percentage of B-1b cells (Cook et al. 1996). The loss of B-2 cells may explain why alcoholics often cannot respond adequately to new antigens. The relative increase in B-1b cells also may lead to autoantibody production, especially of the IgM and IgA classes (which is discussed below).

### Effects on Circulating Immunoglobulin Levels

Igs mediate a critical part of the adaptive immune response. There are five classes of antibodies:

- IgD is present in small amounts in blood and serum and signals naïve B cells to be activated.
- IgM is the first antibody produced during an immune response and is responsible for agglutination and antibody-dependent cytotoxicity.
- IgG is abundant in blood, lymph fluid, cerebrospinal fluid, and peritoneal fluid and can activate the complement system to facilitate phagocytosis of microorganisms.
- IgA is present primarily in mucosal secretions and prevents pathogens from attaching to and penetrating epithelial surfaces.
- IgE is widely found in the lungs, skin, and mucous membranes and plays an important role in allergic, anti-parasitic, and type 1-hypersensitivity responses.

Although chronic alcohol consumption leads to reduced B-cell frequency, it also results in increased production of Igs. Alcoholic patients with ALD (i.e., hepatic fibrosis or cirrhosis) who had consumed the equivalent of 10 oz. of 100-proof ethanol/day for 10 years showed elevated IgA and IgG levels compared with controls or alcoholics without ALD (Smith et al. 1980). Similarly, both IgA and IgM levels were increased in heavy drinkers (90 to 249 drinks/month) compared with light (<9 drinks/month) or moderate drinkers (30 to 89 drinks/month) (Mili et al. 1992). Other studies determined an increase in IgE levels with chronic alcohol consumption (>100g/day for a minimum of 5 years), which was associated with an increased prevalence of pollen allergies (Gonzalez-Quintela et al. 1999, 2003; Hallgren and Lundin 1983). Furthermore, IgE levels decreased gradually with length of abstinence, suggesting a strong correlation between alcohol consumption and IgE serum levels (Gonzalez-Quintela et al. 1995; Hallgren and Lundin 1983).

These clinical observations were confirmed with cultured cells as well as in rodent studies. Treatment of a mouse cell line (i.e., A78-G/A7 hybridoma cells) with different concentrations of ethanol (25, 50, 100, and 200mM) for 48 hours resulted in a linear increase in IgM levels (Muhlbauer et al. 2001). Moreover, spontaneous IgA synthesis by peripheral blood mononuclear cells (PBMCs)— a mixed population of various white blood cells that also includes B cells—was higher in PBMCs isolated from alcoholic patients with liver disease compared with controls (Wands et al. 1981). IgA concentrations also were increased in a layer (i.e., the lamina propria) of the mucous membranes lining the intestine of adult female Wistar rats after acute ethanol administration (4g/kg intraperitoneally) for 30 minutes (Budec et al. 2007). Recent studies suggest that the increase in IgA levels may be mediated by an ethanol-induced elevation of the enzyme neuronal nitric oxide synthase (nNOS) in the animals’ intestine, because inhibition of nNOS before ethanol injection suppressed the IgA increase (Budec et al. 2013). However, additional studies are needed to fully uncover the mechanisms that underlie increased Ig production while B-cell numbers are reduced. Potential mechanisms include increased Ig production by mature B cells (i.e., plasma cells), which have not yet been examined, and increased levels of antigens in the body, either because of higher rates of infection or potentially because of higher production of antigens derived from the body itself (i.e., autoantigens) as a result of organ damage.

Interestingly, ALD patients have increased concentrations (i.e., titers) of circulating antibodies directed against liver-specific autoantigens (McFarlane 2000). Thus, patients with active alcohol-induced hepatitis have antibodies directed against liver-specific membrane lipoprotein, with titers correlating with disease severity (McFarlane 1984). Antibodies against the ethanol-metabolizing enzyme alcohol dehydrogenase also have been found in 50 percent of patients with ALD and were associated with alcoholic hepatitis (Ma et al. 1997). Furthermore, anti-phospholipid antibodies could be observed in up to 80 percent of patients with alcoholic hepatitis or cirrhosis, as well as in heavy drinkers (>80g/day for more than 1 year) with milder liver damage (Chedid et al. 1994). Finally, about 70 percent of patients with advanced ALD have elevated levels of antibodies directed against compounds formed in the body during metabolic processes (i.e., lipid peroxidation) that occur as a result of alcohol-induced ROS production (Mottaran et al. 2002). These compounds, which include malondialdehyde, 4-hydroxynonenal, and lipid hydroperoxides, are readily detectable in the serum and liver of ALD patients and alcohol-fed rodents (Albano 2006). These observations suggest that ethanol-induced organ damage could stimulate auto-antibody production, leading to overall increased concentration of circulating antibodies.

Additional studies conducted with mice indicate that the development of antibodies against antigens generated as a result of lipid peroxidation is associated with the hepatic expression of proinflammatory cytokines and development of fatty liver (i.e., steatohepatitis) (Ronis et al. 2008). Similarly, studies in humans have shown that elevated levels of antibodies directed against lipid-peroxidation products are associated with elevated levels of the proinflammatory cytokine tumor necrosis factor-α in the blood (Vidali et al. 2008). The exact mechanisms by which these autoantibodies lead to increased production of proinflammatory factors remain to be elucidated. It is possible that antibody-mediated opsonization and tissue destruction result in inflammatory cytokine production by various immune cells that ingest the marked antigens (i.e., phagocytic cells or natural killer [NK] cells).^2^

**Figure 2:**
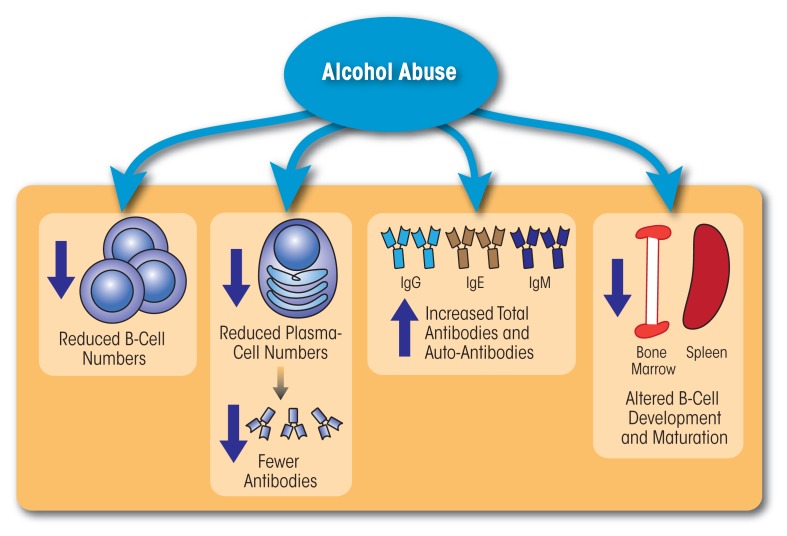
Alcohol abuse impairs both the number and function of B cells. Chronic alcohol consumption reduces B-cell numbers, decreases antigen-specific antibody responses, increases the production of auto-antibodies, and interferes with B-cell development and maturation.

Together, the above studies demonstrate that chronic alcohol consumption simultaneously is associated with reduced B-cell numbers and increased Ig levels, including Igs directed against liver autoantigens and byproducts of oxidative damage (see figure 2). The presence of these antibodies contributes to increased production of inflammatory cytokines, which in turn may accelerate and/or exacerbate liver damage.

## Impact of AUD on Lymphocyte Development

### Effects on Thymocytes

Thymocytes are progenitor cells in the thymus that entered a selection and maturation process called thymopoiesis to become T cells. Alcohol exposure can interfere with this process. Thus, when pregnant female C57BL/6J mice consumed a liquid diet in which 25 percent of the calories were derived from ethanol from gestational day 1 to day 18, the offspring exhibited reduced thymocyte numbers (Ewald 1989; Ewald and Frost 1987; Ewald and Walden 1988). This reduction in thymocyte numbers may be mediated by ethanol-associated activation of the hypothalamic–adrenal–pituitary axis and increased glucocorticoid levels (Hiramatsu and Nisula 1989).^3^ Glucocorticoids long have been recognized as a potential cause for shrinkage (i.e., atrophy) of the thymus through induction of apotopsis (Reichert et al. 1986). This hypothesis is supported by findings that a single dose of ethanol results in increased levels of endogenous glucocorticoids and thymic atrophy (Han et al. 1993). Furthermore, administration of a glucocorticoid antagonist can block thymic atrophy and DNA fragmentation indicative of apoptosis in 8- to 12-week-old female mice consuming a solution of 20 percent ethanol in water (Han et al. 1993). However, another study showed that ethanol-fed animals whose adrenal glands had been removed (i.e., which had been adrenalectomized) and which therefore could no longer produce glucocorticoids still had fewer thymocytes than control adrenalectomized animals (Jerrells et al. 1990). This suggests that glucocorticoids are responsible for some, but not all, of the ethanol-induced thymic dysfunction.

Alcohol also activates an enzyme acting at the thymocyte membrane called adenylate cyclase, which increases the intracellular concentration of cyclic AMP (Atkinson et al. 1977). cAMP has multiple regulatory functions in the cell, and increased cAMP levels can stimulate DNA fragmentation, leading to thymocyte apoptosis (McConkey et al. 1990). In studies conducted in vitro using a controlled model of thymocyte differentiation known as fetal thymus organ culture, exposure to 0.2 or 0.4 percent ethanol for 5 days resulted in generation of fewer total thymocytes and increased thymocyte apotopsis in a dose-dependent manner compared with control cultures (Bray et al. 1993). Finally, exposure to ethanol concentrations of 0.4 to 2 percent had a more profound effect on apoptosis of cultured thymocytes than on mature T cells (Slukvin and Jerrells 1995). All of these studies demonstrate that ethanol interferes with normal thymocyte function and maturation into T cells in a variety of ways.

### Effects on B-Cell Development

Numerous analyses also have evaluated the effects of ethanol exposure on the development of B cells. As described above for thymopoiesis, the offspring of pregnant mice that from gestational day 1 to day 18 consumed a liquid diet in which 25 percent of calories were derived from ethanol exhibited decreased numbers of both immature and mature B cells in the spleens directly after birth. Moreover, these B-cell subpopulations did not recover to normal levels until 3 to 4 weeks of life (Moscatello et al. 1999; Wolcott et al. 1995). Other studies were conducted using a precursor cell type called oligoclonal-neonatal-progenitor (ONP) cells, which in vitro can differentiate either into B lymphocytes or into other white and red blood cells (i.e., myeloid cells), depending on the cytokines to which they are exposed. ONP cells isolated from newborn mice that had been exposed to alcohol in utero demonstrated a greatly reduced capacity to respond to interleukin (IL)-7 and commit to the B lineage, whereas their response to the growth factor granulocyte–monocyte colony-stimulating factor (GM-CSF) and commitment to the myeloid lineage was not affected (Wang et al. 2006). Similarly, ONP cells isolated from newborn mice and cultured in vitro in the presence of 100 mM ethanol for 12 days failed to respond to IL-7 and commit to the B lineage, suggesting intrinsic defects (Wang et al. 2011). Additional investigations demonstrated that alcohol affects ONP cell differentiation into B lineage at a late stage by down-regulating the expression of several transcription factors (e.g., EBF and PAX5) and cytokine receptors, such as the IL-7 receptor (IL-7Ra) (Wang et al. 2009).

As described earlier for adult humans, alcohol can lead to increases in Ig levels during development, even if the numbers of mature B cells decrease. Thus, maternal alcohol consumption during pregnancy (12 mg/week for most of the pregnancy) increased IgE levels in the umbilical cord blood of the infants (Bjerke et al. 1994).

Taken together, all these findings suggest that in utero exposure to ethanol may increase the risk for infections during early childhood or adulthood as a result of alcohol-induced defects in B-cell and T-cell development. Indeed, in utero exposure to ethanol resulted in a significant reduction in T-cell and B-cell responses to various antigens that did not recover to control levels until 4 to 5 weeks of life. In contrast, ethanol exposure did not significantly affect the development of the lytic functions of NK cells (Wolcott et al. 1995).

## Impact of AUD on Adaptive Immune Responses

### Responses to Infections

Alcohol abuse has been associated with increased incidence and severity of community-acquired pneumonia (Happel and Nelson 2005; Zhang et al. 2002), HIV infection (Mbulaiteye et al. 2000; Rasch et al. 2000; Stein et al. 2000; Welch 2000), HCV infection (Seronello et al. 2010; Zhang et al. 2003), and infection with *Mycobacterium tuberculosis* (Hedemark et al. 1995; Hudolin 1975; Kline et al. 1995; Panic and Panic 2001; Sabot and Vendrame 1969). This increased susceptibility could be caused by alcohol-induced alterations in lymphocyte numbers and function or by AUD-related enhanced behavioral or environmental exposure to these pathogens.

Analyses of animal models can help delineate the contribution of behavioral and immunological changes to the increased susceptibility to infection. Indeed, experiments in a mouse model of influenza A infection showed that animals that had consumed 18 to 20 percent ethanol for 4 to 8 weeks exhibited an impaired influenza-specific CD8 T-cell response. Specifically, mice in the ethanol group exhibited a decrease in the number of influenza-specific CD8 T cells (Meyerholz et al. 2008).^4^ Influenza A virus infections increasingly are recognized as an important agent in community-acquired pneumonia. Because influenza-specific effector CD8 T cells play a central role in the elimination of influenza-infected cells (Epstein et al. 1998), a reduced T-cell response could lead to increases in the incidence and severity of community-acquired pneumonia (Horimoto and Kawaoka 2005). Finally, adult mice exposed to ethanol only during gestation and nursing exhibited increased influenza-associated morbidity and mortality, increased numbers of virus particles in the lungs, and decreased numbers of both B cells and influenza-specific CD8 T cells in the lungs following influenza infection (McGill et al. 2009).

Researchers also have investigated the molecular and cellular mechanisms underlying increased susceptibility to HIV associated with chronic drinking using animal models. In one approach, rhesus macaques were administered either alcohol or a sugar solution with the same calorie content directly into the stomach. When both groups of animals were infected with the primate equivalent of HIV (i.e., simian immunodeficiency virus [SIV]) by the rectal route, higher SIV loads were observed in the alcohol-consuming animals. In addition, alcohol-consuming animals exhibited a higher CD4:CD8 T-cell ratio in part of the intestine (i.e., the duodenum) compared with control animals (Poonia et al. 2006). Because intestinal CD4 T cells are the major target cells in HIV and SIV infections (Veazey et al. 2001), an increased percentage of CD4 T cells in the gut of alcohol-consuming macaques could be the reason for the higher SIV loads observed in these animals (Poonia et al. 2006). In addition, CD8 T-cell responses play a critical role in controlling HIV infections and eliminating infected cells; therefore, the decrease in CD8 T cells could lead to impairment in anti-HIV responses (Betts et al. 2006).

The increased susceptibility to *M. tuberculosis* was confirmed in a mouse study where consumption of a liquid ethanol diet for 9 weeks (serum alcohol levels = 39 mg/dL) resulted in significantly higher bacterial burden in the lung (Mason et al. 2004). Further analyses also identified blunted CD4 T-cell responses (i.e., reduced proliferation as well as IFN-γ and IL-2 production by the cells) as well as decreased CD8 T-cell numbers in draining lymph nodes of alcohol-consuming mice compared with control mice (Porretta et al. 2012).

### Responses to Vaccination

Because alcoholics are at increased risk for hepatitis B (HepB) infections, immunization with a HepB vaccine is recommended. However, the magnitude of the response to the vaccination (i.e., the production of antibodies) is lower in alcoholics compared with nonalcoholic control subjects (Nalpas et al. 1993), patients with other drug dependencies (Hagedorn et al. 2010), or patients with chronic liver disease caused by HCV or unknown causes (i.e., cryptogenic liver disease) (Roni et al. 2013), with the lowest responses found in alcoholics with liver disease. Another study (Rosman et al. 1997) demonstrated that the impaired antibody response in alcoholic patients (i.e., with consumption levels of 230 ± 16 g/day ethanol for 26.4 ± 1.8 years) can be improved by doubling the dose of HepB vaccine from 10 μg to 20 μg at 0, 1, and 6 months. Similar results also were obtained in animal models. Thus, mice that were chronically fed ethanol generated a weaker antibody response following vaccination with HCV compared with control mice (Encke and Wands 2000). Abstinence partially restored antibody responses against hepatitis antigens in a mouse model (Encke and Wands 2000).

Additional studies in rodents assessed the effects of alcohol on the effectiveness of bacillus Calmette-Guérin (BCG) vaccination, which protects against tuberculosis. The studies found that when animals consumed ethanol before BCG vaccination, they were not protected against a subsequent pulmonary challenge with *M. tuberculosis*. In contrast, mice that consumed ethanol after the BCG vaccination were protected against a subsequent *M. tuberculosis* challenge (Porretta et al. 2012). Taken together, these data suggest that chronic ethanol exposure interferes with immunity to new antigens but not with immunity established before alcohol consumption.

### Cancer Risk

Alcohol-related alterations of immune surveillance also have been implicated in the development of cancer (Poschl and Seitz 2004). Reduced cell-mediated immunity was proposed as a potential explanation for the high incidence of head and neck cancer observed in alcoholic patients (Lundy et al. 1975). However, these studies are difficult to interpret, because several factors affect antitumor immunity in human alcoholics, including malnutrition, vitamin deficiencies, and liver cirrhosis. The impact of alcohol on NK cells, which are the first responders against tumor-forming cells, has been investigated in mouse models. Those studies showed decreased cytolytic activity of NK cells in C57BL/6 mice consuming 20 percent ethanol for 4 weeks; however, no differences existed in the metastasis of B16-BL6 melanoma cells in alcohol-consuming and control animals (Meadows et al. 1993). Another study using different tumor cells (i.e., MADB106 mammary adenocarcinoma cells) demonstrated that ethanol administration 1 hour before tumor inoculation suppressed NK-dependent destruction of tumor cells, resulting in a 10-fold increase in the number of lung metastases in Fischer 344 rats (Ben-Eliyahu et al. 1996). The presence of ethanol in an in vitro culture of spleen cells also suppressed NK cell cytotoxic activity against MADB106 tumor cells (Yirmiya et al. 1992).

### Delayed-Type Hypersensitivity

Another aspect of cell-mediated immunity that is affected by ethanol consumption is the delayed-type hypersensitivity (DTH) response. DTH refers to a cutaneous T-cell–mediated inflammatory reaction that takes 2 to 3 days to develop. It is mediated by CD4 T helper cells, specifically the Th1 subpopulation. The data on alcohol-induced alterations in DTH responses are limited. One early study (Lundy et al. 1975) showed defects in cell-mediated immunity in male alcoholic patients admitted for detoxification, in response both to a new antigen and to an antigen to which they had previously been exposed. A more recent study (Smith et al. 2004) reported that a negative correlation existed between the amount of alcohol consumed by the participants and the size of DTH skin test responses to a specific antigen (i.e., keyhole limpet hemocyanin). Similar results were described in rodent models. For instance, genetically modified BALB/c mice that carried a TCR specific for the ovalbumin peptide and were fed a diet containing 30 percent ethanol-derived calories exhibited decreased antigen-specific Th1 responses (Waltenbaugh et al. 1998). Similarly, C57BL6 mice fed a liquid diet in which ethanol provided 27 percent of the total calories generated significantly decreased DTH responses to a T-cell–dependent antigen (i.e., sheep red blood cells) (Jayasinghe et al. 1992). The reduced DTH response and accompanying decrease in IL-12 and IFN-γ cytokine production are thought to result in part from ethanol-mediated depletion of the antioxidant glutathione in antigen-presenting cells (Peterson et al. 1998).

## Effects of Moderate Ethanol Consumption on Adaptive Immunity

The discussion in the preceding sections centered primarily on the effects of chronic alcohol abuse on the immune system. In contrast with these generally detrimental effects, data from several studies suggest that moderate alcohol consumption may exert beneficial effects on the adaptive immune system. For example, healthy volunteers who had a 30-day alcohol abstinence period before drinking moderately (i.e., 330 mL beer per day for women and 660 mL for men) for 30 days showed significant increases in a variety of variables associated with adaptive immune responses (e.g., CD3 subsets; secretion of IL-2, IL-4, IL-10, and IFN-γ by mitogen-stimulated PBMCs; and levels of IgG, IgM, and IgA in the blood) (Romeo et al. 2007).

Several studies also have reported improved responses to vaccination and infection in both humans and animal models of moderate alcohol consumption. A study exploring the impact of alcohol consumption on the incidence of colds among 391 subjects intentionally exposed to 5 different respiratory viruses showed that moderate alcohol consumption (i.e., 1 to 2 drinks/day) was associated with decreased incidence of colds in nonsmokers (Cohen et al. 1993). Similarly, people who consumed a moderate amount of wine (i.e., 3.5 glasses), and especially red wine, had a reduced incidence of the common cold compared with nondrinkers (Takkouche et al. 2002). In a rat model, low to moderate ethanol doses resulted in a greater delayed cutaneous hypersensitivity response and improved clearance of *Mycobacterium bovis,* whereas high ethanol doses were associated with a reduced response and decreased bacterial clearance (Mendenhall et al. 1997). Finally, in a rhesus macaque model, animals that voluntarily consumed moderate amounts of ethanol (1.3 to 2.3 g/kg/day) showed an improved response to a vaccine to which the animals had been exposed before (i.e., recall vaccine response) compared with controls (Messaoudi et al. 2013).

The mechanisms by which moderate alcohol consumption might exert these beneficial effects are only beginning to emerge. In a study examining the impact of moderate alcohol consumption on gene-expression patterns in blood cells (Joosten et al. 2012), young men consumed either 100 mL vodka with 200 mL orange juice or only orange juice daily during dinner for 4 weeks. After this period, the moderate-drinking participants exhibited down-regulation of a transcription factor (i.e., NF-Kappa B), modulation of pathways of antigen presentation, altered B- and T-cell receptor signaling, and reduced IL-15. Furthermore, the plasma levels of various proinflammatory signaling molecules (e.g., positive acute phase protein ferritin, α1-antitrypsin, and cytokines such as an IL-1 receptor agonist and IL-18) were significantly reduced, whereas anti-inflammatory proteins such as adiponectin were increased after moderate alcohol consumption (Joosten et al. 2012).

## Summary

Studies over the last 30 years have clearly demonstrated that chronic ethanol abuse impairs the functions of both T cells and B cells. Chronic alcohol consumption results in lymphopenia with a loss in circulating T cells and B cells. The decrease in T cells is accompanied by increased homeostatic proliferation, which in turn leads to increased T-cell differentiation, activation, and conversion to the memory phenotype. Impairment in T-cell recruitment also was observed in mouse models of chronic alcohol exposure. Despite reduced B-cell numbers, people with AUD exhibit increased serum concentration of IgA, IgG, and IgE. This increase in circulating Igs correlates with increased levels of antibodies directed against liver antigens and byproducts of oxidative damage. Finally, alcohol exposure in utero significantly interferes with the development of T cells and B cells, which ultimately might increase risk for infections during adulthood. In contrast to the devastating effects of chronic alcohol abuse, a few studies have shown that moderate alcohol consumption increases the number of T cells; improves T-cell cytokine production; and enhances immune response to vaccines in humans, nonhuman primates, and rodents.

The molecular mechanisms underlying the dose-dependent impact of ethanol on immunity remain poorly understood. Most studies have been carried out in vitro using primary cells or cell lines in the presence or absence of ethanol. The use of cultured cells presents several advantages, such as the ability to precisely control the amount and duration of ethanol exposure, the relatively low cost, the ease of culturing large quantities of cells, and the ease of manipulating nutrients in the media and regulating gene expression. However, in vitro studies alone cannot reveal the underlying mechanisms of immune modulation by ethanol, because immune cells carry out their functions in a multicellular environment. Therefore, in vivo studies also are necessary. These studies frequently use rodent models, which allow researchers to use an abundance of reagents to characterize the immune response and to access genetically modified animals (i.e., transgenic and knockout strains) that facilitate mechanistic studies. However, alcohol consumption is not voluntary in these models. Thus, there is a pressing need to conduct additional studies using clinical samples or animal models that more faithfully mirror the complexity of human alcohol consumption, metabolism, and immune responses. Macaques are genetically diverse and, like humans, consume alcohol voluntarily and exhibit a wide spectrum of drinking patterns. Additionally, their long lifespan facilitates the study of the effects of long-term alcohol consumption, and their large size allows simultaneous longitudinal sampling from various body tissues and organs that harbor immune cells (e.g., blood, lung, and gut). The tools available to study immunity in nonhuman primate models also are becoming more sophisticated. However, the high cost associated with nonhuman primate models remains an obstacle for large-scale studies.

**Figure 3:**
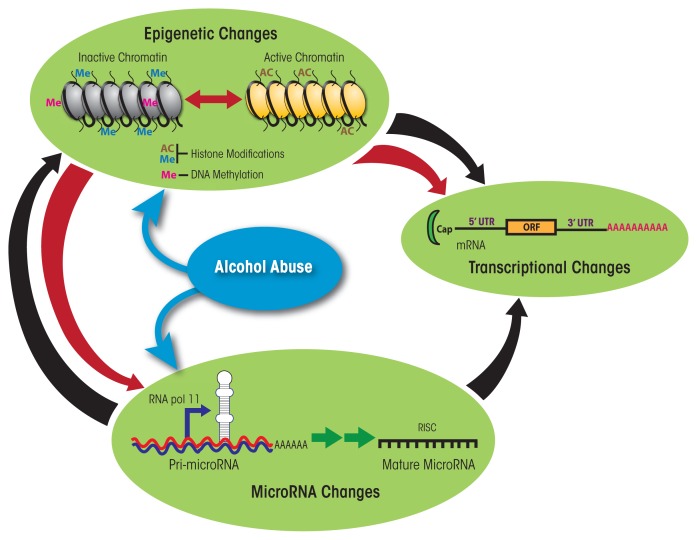
Alcohol modulates gene expression—that is, the generation of mRNAs and, ultimately, functional proteins from the DNA template—through changes in noncoding microRNA (miRNA) levels and epigenetic modifications. These epigenetic modifications, which include methylation of the DNA as well as modifications (e.g., acetylation and methylation) of the histone proteins around which the DNA is wound, determine whether the complex of DNA and histones (i.e., the chromatin) is in an active or inactive conformation. Such epigenetic changes can promote (red arrow) or inhibit (black arrow) the expression of mRNAs as well as promote the expression of certain miRNAs (including the processing of precursor molecules called pri-micro RNA into mature miRNA). Conversely, miRNAs can inhibit the actions of the methylation machinery and expression of proteins involved in histone modifications as well as can interfere with the transcription of mRNAs. NOTE: 3′ UTR = 3′ untranslated region; 5′ UTR = 5′ untranslated region; ORF = open reading frame; RISC = RNA-induced silencing complex, a complex of miRNA and proteins.

Future studies should leverage the different models to uncover the molecular mechanisms underlying the dose-dependent impact of alcohol on immune function by investigating changes in gene expression patterns (Mayfield and Harris 2009). Such approaches should also investigate the contributions of noncoding RNAs, such as microRNAs (miRNAs), and epigenetic modifications, which are known to regulate gene expression patterns (Curtis et al. 2013; Sato et al. 2011). miRNAs are small, single-stranded, noncoding RNAs that bind within one end of the target genes and prevent the generation of functional proteins from these genes by either destabilizing the mRNAs generated from the genes, preventing the translation of the genetic information in the mRNA into a protein, or both (Ambros 2004; Bartel 2004; Filipowicz et al. 2008). A single miRNA can target hundreds of mRNA transcripts, and a single mRNA transcript simultaneously can be targeted by more than one miRNA, ensuring fine-tuned and/or redundant control over a large number of biological functions. Epigenetic modifications are chemical changes that occur within a genome without changing the DNA sequence. These changes include direct addition of a methyl group to DNA (i.e., DNA methylation) or chemical modifications of the proteins (i.e., histones) around which DNA is wrapped, such as acetylation, methylation, and phosphorylation (Holliday 2006; Hsieh and Gage 2005; Murrell et al. 2005). Both regulatory mechanisms related to miRNA and epigenetic mechanisms are interrelated (see figure 3). Thus, several miRNAs themselves are regulated epigenetically but also are capable of targeting genes that control epigenetic pathways (e.g., polycomb group-related genes and histone deacetylase). Studies have identified ethanol-mediated changes in both miRNA abundance (Miranda et al. 2010; Pietrzykowski 2010) and epigenetic modifications within PBMCs (Biermann et al. 2009; Bleich and Hillemacher 2009; Bonsch et al. 2006). Other investigators have described ethanol-induced epigenetic modifications (i.e., alterations in histone acetyltransferases and histone deacetylases) in liver cells (i.e., hepatocytes) in rodent models of binge drinking and ALD (Bardag-Gorce et al. 2007; Choudhury et al. 2010; Park et al. 2005; You et al. 2008). However, very few studies have examined ethanol-induced changes in gene expression and regulation within specific immune-cell subsets. Moreover, none of the studies have conducted a comprehensive integrated analysis of mRNA, miRNA, and epigenetic expression patterns in the same cell(s) before and after alcohol consumption. Integrating gene expression patterns with gene regulation could reveal novel insight into specific pathways that are dysregulated with alcohol abuse and could explain the increased susceptibility to infection. These insights could lead to interventions to restore immunity, such as reversing changes in histone modifications and DNA methylation patterns or modulating expression levels of miRNAs. In addition, such studies could reveal the pathways that are modified by moderate alcohol consumption to enhance immune response to vaccination.

## Footnotes

1 T-cell activation was assessed by measuring the expression of human leukocyte antigen (HLA)-DR on the patient’s CD8 cells. HLAs are proteins found on the surface of various cells that present antigens to the TCR on T cells to induce an immune response.

2 Opsonization is a process by which a pathogen or other antigen is covered with antibodies and thereby marked for ingestion and destruction by other immune cells (i.e., phagocytic cells).

3 The hypothalamic–adrenal–pituitary axis is a hormonal system that primarily is involved in the stress response. Activation of this system culminates in the production and release of corticosteroid (i.e., cortisol in humans and corticosterone in rodents) from the adrenal glands, which then act on various tissues to mediate the stress response.

4 Similarly, chronic consumption of 18 percent ethanol in water for 31 weeks resulted in impaired antigen-specific CD8 T-cell responses following inoculation with *Listeria monocytogenes* (Gurung et al. 2009).
